# Predictive role of *ARID1A* and *B2M* mutations and the antigen presentation pathway in the efficacy of definitive chemoradiotherapy for cervical cancer

**DOI:** 10.1093/oncolo/oyaf133

**Published:** 2025-06-19

**Authors:** Chenjing Zhu, Zhen Gong, Ping Yin, Jian Huang, Dan He, Biqing Zhu, Yaqin Wu, Hairong Wang, Yaru Zhang, Qifan Jing, Jiani C Yin, Yue Li, Jianyao Liu, Huanhuan Hu, Shuyue Xiao, Zhihua Sun, Hanzi Xu

**Affiliations:** Department of Radiation Oncology, The Affiliated Cancer Hospital of Nanjing Medical University & Jiangsu Cancer Hospital & Jiangsu Institute of Cancer Research, Nanjing 210009, People’s Republic of China; Department of Gynecology, Women’s Hospital of Nanjing Medical University & Nanjing Women and Children’s Healthcare Hospital, Nanjing 210004, People’s Republic of China; Department of Radiation Oncology, The Affiliated Cancer Hospital of Nanjing Medical University & Jiangsu Cancer Hospital & Jiangsu Institute of Cancer Research, Nanjing 210009, People’s Republic of China; Department of Radiation Oncology, The Affiliated Cancer Hospital of Nanjing Medical University & Jiangsu Cancer Hospital & Jiangsu Institute of Cancer Research, Nanjing 210009, People’s Republic of China; Department of Radiation Oncology, The Affiliated Cancer Hospital of Nanjing Medical University & Jiangsu Cancer Hospital & Jiangsu Institute of Cancer Research, Nanjing 210009, People’s Republic of China; Department of Radiation Oncology, The Affiliated Cancer Hospital of Nanjing Medical University & Jiangsu Cancer Hospital & Jiangsu Institute of Cancer Research, Nanjing 210009, People’s Republic of China; Department of Radiation Oncology, The Affiliated Cancer Hospital of Nanjing Medical University & Jiangsu Cancer Hospital & Jiangsu Institute of Cancer Research, Nanjing 210009, People’s Republic of China; Department of Radiation Oncology, The Affiliated Cancer Hospital of Nanjing Medical University & Jiangsu Cancer Hospital & Jiangsu Institute of Cancer Research, Nanjing 210009, People’s Republic of China; Nanjing Geneseeq Technology Inc., Nanjing 210000, People’s Republic of China; Nanjing Geneseeq Technology Inc., Nanjing 210000, People’s Republic of China; Nanjing Geneseeq Technology Inc., Nanjing 210000, People’s Republic of China; Department of Radiation Oncology, The Affiliated Cancer Hospital of Nanjing Medical University & Jiangsu Cancer Hospital & Jiangsu Institute of Cancer Research, Nanjing 210009, People’s Republic of China; Department of Radiation Oncology, The Affiliated Cancer Hospital of Nanjing Medical University & Jiangsu Cancer Hospital & Jiangsu Institute of Cancer Research, Nanjing 210009, People’s Republic of China; Department of Gynecology, Women’s Hospital of Nanjing Medical University & Nanjing Women and Children’s Healthcare Hospital, Nanjing 210004, People’s Republic of China; Department of Gynecology, Women’s Hospital of Nanjing Medical University & Nanjing Women and Children’s Healthcare Hospital, Nanjing 210004, People’s Republic of China; Department of Radiation Oncology, The Affiliated Cancer Hospital of Nanjing Medical University & Jiangsu Cancer Hospital & Jiangsu Institute of Cancer Research, Nanjing 210009, People’s Republic of China; Department of Radiation Oncology, The Affiliated Cancer Hospital of Nanjing Medical University & Jiangsu Cancer Hospital & Jiangsu Institute of Cancer Research, Nanjing 210009, People’s Republic of China

**Keywords:** genomic profiling, prognosis, cervical cancer, definitive chemoradiotherapy

## Abstract

**Background and purpose:**

Definitive chemoradiotherapy (dCRT) is the standard treatment for locally advanced cervical cancer (LACC), yet patients experience considerable variability in disease-free survival (DFS). This study aimed to identify molecular biomarkers associated with response to dCRT in cervical cancer.

Materials and methods: We retrospectively analyzed targeted next-generation sequencing data from tumor biopsy samples of 31 patients diagnosed with FIGO stage IIB–IVA LACC. Genetic alterations in cancer-related genes and pathways were assessed to determine associations with DFS. Immune cell infiltration and gene expression were analyzed using data from The Cancer Genome Atlas.

**Results:**

Genetic alterations were frequently detected in *PIK3CA* (45.2%), *EP300* (25.8%), *RB1* (19.4%), *FBXW7* (19.4%), and *FAT1* (16.1%). Multivariate analysis identified mutations in *ARID1A* and *B2M* as independent predictors of poor DFS. Alterations in the antigen processing and presentation pathway were also associated with reduced survival rates. Patients with *ARID1A* and *B2M* mutations exhibited decreased immune cell infiltration and impaired antigen presentation, indicating a compromised immune response.

**Conclusion:**

*ARID1A* and *B2M* mutations may serve as potential biomarkers for predicting treatment outcomes in cervical cancer patients undergoing dCRT. Testing for these mutations could help identify patients at higher risk of poor outcomes, guiding personalized treatment strategies to improve survival rates.

**Implications for practice**

This study identifies 2 genetic mutations, *ARID1A* and *B2M*, that are associated with poorer outcomes in patients with locally advanced cervical cancer undergoing standard chemoradiotherapy. The presence of these mutations may suppress the immune system’s response to cancer, reducing the effectiveness of treatment. Screening for these mutations can help clinicians predict which patients are less likely to benefit from conventional therapy. This insight enables personalized treatment strategies, such as incorporating immunotherapy, to improve patient outcomes. Recognizing these genetic markers is a vital step toward enhancing survival rates and tailoring care for patients with this challenging disease.

## Introduction

Cervical cancer remains one of the leading causes of maternal morbidity and mortality among women worldwide.^1^ In 2020, it was estimated that 604,000 women were diagnosed with cervical cancer, and 342,000 women died from the disease globally.^2^ High-risk human papillomavirus (HPV) infection is the primary etiological factor, with additional risk factors including sexually transmitted infections (eg, HIV and *Chlamydia trachomatis*), smoking, high parity, and prolonged use of oral contraceptives.^3,4^ These factors collectively contribute to disease progression.

For patients with locally advanced cervical cancer (LACC), definitive chemoradiotherapy (dCRT), which combines platinum-based chemotherapy and radiotherapy, remains the standard treatment.^5,6^ Despite treatment advances, many patients experience disease progression or relapse,^7,8^ highlighting the need for biomarkers to predict treatment response and disease-free survival (DFS). New therapies, including immune checkpoint inhibitors, ribonucleotide reductase inhibitors, and poly (ADP-ribose) polymerase inhibitors, show promise in treating metastatic or recurrent disease, though patient responses are highly variable.^9-12^ This variability reinforces the need for new biomarkers for personalized treatment.

Our study sought to investigate the genomic and immunological landscape of cervical cancer patients undergoing dCRT, with the goal of identifying potential biomarkers that may predict treatment efficacy and DFS. By elucidating these factors, we aim to offer insights that could lead to more effective treatment protocols and improved outcomes for patients with LACC.

## Materials and methods

### Subject selection

This study included 31 patients with cervical cancer who were treated with dCRT at Jiangsu Cancer Hospital between September 2019 and March 2023. This study was approved by the Ethics Committee of Jiangsu Cancer Hospital (No. 2023K-K096), and all participants provided written informed consent prior to enrollment.

The inclusion criteria were as follows: (1) histologically confirmed cervical cancer; (2) disease classified as stage IIB-IVA according to the Federation of Gynecology and Obstetrics (FIGO) staging system for cervical cancer^13^; (3) dCRT as primary treatment; (4) an Eastern Cooperative Oncology Group (ECOG) performance status score of 0 to 2; and (5) availability of complete clinical data. The exclusion criteria were as follows: (1) age over 70 years old; (2) severe or uncontrolled comorbidities or secondary malignancies; (3) prior anti-tumor treatments, including surgery, targeted therapy, immunotherapy, or radiotherapy; and (4) refusal to participate in the study.

### General information and clinical data

General information and clinical data were collected for each patient, including age, FIGO stage, histological type, and pathological grade. Tumor size at diagnosis and HPV infection status were recorded. HPV infection status was determined using the HPV Nucleic Acid Detection and Genotyping Kit (PCR-capillary electrophoresis fragment analysis; product no. 1060037; HEALTH GENETECH), according to the manufacturer’s protocol. Radiographic assessments, including computed tomography (CT), magnetic resonance imaging (MRI), and positron emission tomography-computed tomography (PET-CT), were obtained to evaluate disease extent. Additional clinical information, such as the date of diagnosis, treatment regimen, disease progression status, and survival outcomes, was also collected for subsequent analyses.

### Treatment

The clinical target volume (CTV) encompassed the gross tumor, uterus, parametrium, portions of the vagina, and pelvic lymphatic regions, including the common iliac, external iliac, internal iliac, obturator, and presacral lymph nodes. In patients with para-aortic lymph node metastasis, extended-field external beam radiotherapy (EBRT) was administered to cover the para-aortic region. Metastatic lymph nodes were classified as gross tumor volume (GTVnd). An isotropic expansion of 6-8 mm from the CTV defined the planning clinical target volume (PCTV), and a 5 mm margin was added to the GTVnd to create the planning gross tumor volume (PGTVnd). Patients underwent pelvic EBRT with a total dose of 45-50 Gy, delivered in daily fractions of 1.8 Gy or 2.0 Gy, 5 times a week, concurrently with a platinum-based chemotherapy regimen. Positive lymph nodes received an additional boost dose of 10 to 15 Gy. Following the completion of EBRT, patients received high-dose-rate (HDR) intracavitary brachytherapy using an Iridium-192 radioactive source, administered in 5 fractions (1-2 fractions per week) for a total dose of 30.0 Gy.

### Follow-up and evaluation

Follow-up commenced 1 month after completing radiotherapy and continued quarterly during the first year, followed by every 3-6 months thereafter. The DFS of this study was defined as the interval from diagnosis to loco-regional relapse, distant metastasis, or death from any cause, whichever occurred first. Evaluations included gynecological examination, ultrasound, CT, MRI, and PET-CT. Disease response was assessed using the Response Evaluation Criteria in Solid Tumors (RECIST) guideline, version 1.1. The last follow-up was performed in February 2024. The mean follow-up duration was 12.5 months, with a maximum of 43 months.

### DNA extraction and sequencing library construction

Formalin-fixed paraffin-embedded (FFPE) tumor samples were processed for targeted next-generation sequencing (NGS) at a Clinical Laboratory Improvement Amendments (CLIA)-certified, College of American Pathologists (CAP)-accredited test laboratory (Nanjing Geneseeq Technology Inc., Nanjing, China), using a pan-cancer gene panel (GeneseeqPrime, Geneseeq Technology Inc.). Tumor samples were sequenced if the tumor cell content was ≥20%. DNA extraction, library construction, and targeted capture enrichment followed established standard protocols.^14,15^

DNA was extracted using the QIAamp DNA FFPE Tissue Kit (Qiagen). Libraries were constructed using the KAPA Hyper Prep Kit (KAPA Biosystems). Targeted capture was performed using custom xGen Lockdown Probes (Integrated DNA Technologies), designed to enrich 437 cancer-related genes. Hybridization capture was carried out using Dynabeads M-270 (Life Technologies). Libraries were amplified using Illumina p5 (5′-AAT GAT ACG GCG ACC GA-3′) and p7 primers (5’-CAA GCA GAA GAC GGC ATA CGA GAT-3′) in KAPA HiFi HotStart ReadyMix, and purified using Agencourt AMPure XP beads.

Library quantification was performed using the KAPA Library Quantification Kit, and fragment sizing was validated on the Bioanalyzer 2100 (Agilent Technologies).^16,17^ Sequencing was conducted on the Illumina HiSeq4000 NGS platform (Illumina), following the manufacturer’s guidelines.

### Mutation calling

Sequence reads were processed using bcl2fastq V.2.16.0.10 (Illumina) to generate FASTQ files, followed by quality filtering with Trimmomatic.^17,18^ High-quality reads were aligned to the human genome (hg19, GRCh37) using the Burrows-Wheeler Aligner (BWA) V.0.7.12.^17,19^ BAM files were generated and sorted using Picard V.1.119; local realignment around indels and base quality recalibration were performed with the Genome Analysis Toolkit (GATK) V.3.4-0.^17^

Single-nucleotide variations (SNVs) and indels were called using VarScan2,^20^ with a minimum variant allele frequency (VAF) of 0.01 and a p-value threshold of 0.05. Variants were annotated with ANNOVAR and manually validated using the Integrative Genomics Viewer. Filtering criteria included: (1) minimum read depth of 20; (2) minimum base quality of 15; (3) at least 5 supporting reads; (4) presence on both DNA strands; (5) strand bias below 10%; (6) allele frequency < 1% in the 1000 Genomes or ExAC database^21^; and (7) exclusion of recurrent sequencing artifacts. The sequencing assay was validated according to CAP and CLIA standards, with a detection limit set at 1% VAF.^17^

Tumor mutation burden (TMB) was calculated as the total number of somatic synonymous mutations per megabase of the coding region, excluding hotspots and fusions.^22,23^ The chromosomal instability (CIN) score was determined by assessing the proportion of DNA segments with a log2 ratio deviating by more than ± 0.2 across the genome.^24^

### Immune cell infiltration and gene expression analysis of TCGA-CCA cohort

RNA sequencing data from The Cancer Genome Atlas cervical cancer (TCGA-CCA) cohort (*N* = 306) were analyzed, focusing on samples harboring mutations in *ARID1A*, *B2M*, or alterations in the antigen processing and presentation pathway. Immune cell infiltration was estimated using XCELL^25^ from TIMER2.0 website (http://timer.cistrome.org/), which provides immune and stromal scores reflecting cell composition within the tumor microenvironment, inversely correlating with tumor purity.^25^ The mRNA expression level of immune-related genes,^26^ including antigen presentation, cell adhesion, co-inhibitor, co-stimulator, ligand, receptor, and others, was compared. The 50 HALLMARK gene sets were downloaded from the MSigDB.^27^Gene set enrichment analysis (GSEA)^28^ used GSEA v4.3.0 software to analyze and compare the variations in pathway activities from the 50 HALLMARK gene sets, with a false discovery rate (FDR) < 0.25 and an adjusted p < 0.05 as significance thresholds.

### Statistical analysis

Statistical analyses were performed using Cox proportional hazards models to evaluate associations between clinical characteristics and DFS. Survival analyses utilized Kaplan-Meier estimations, and comparisons were made using the log-rank test. Hazard ratios (HRs) were calculated using Cox models. A 2-sided *P*-value of less than 0.05 was considered statistically significant. All statistical analyses were conducted using R software (version 3.5.3).

## Results

### Patient characteristics and treatment outcomes

The clinical characteristics of the 31 cervical cancer patients are outlined in Table 1. The median age at diagnosis was 53 years, ranging from 30 to 69 years. Histopathological analysis identified 26 cases (83.9%) as squamous cell carcinoma (SCC) and 5 cases (16.1%) as adenocarcinoma (ADC). According to FIGO staging, 28 patients (90.3%) were classified as stages IIB-III, and 3 patients (9.7%) were at stage IVA. The TMB of our cohort ranged between 2.1 and 137 (median: 6.2). The CIN score of our cohort ranged between 0.009 and 0.57 (median:0.24). HPV status varied across the cohort: 17 patients (54.8%) tested positive for HPV types 16 or 18, 8 patients (25.8%) were positive for other HPV strains (types 31/35/58/59/68/81), and 6 patients (19.4%) tested negative for HPV. HPV types 16 and 18 are considered high-risk and are typically associated with poorer prognosis.^29^ Thus, these cases were differentiated from those with other HPV types in subsequent analyses.

**Table 1.** Clinical characteristics of cervical cancer patients (*N* = 31).

Characteristics	Total (*N* = 31)
Median age at diagnosis, years	53(30-69)
≤50	10(32.3%)
>50	21(67.7%)
FIGO stage	
IIB-III	28(90.3%)
IVA	3(9.7%)
Histological type	
Squamous cell carcinoma	26(83.9%)
Adenocarcinoma	5(16.1%)
Differentiation	
G2	21(67.7%)
G3	3(9.7%)
G4	7(22.6%)
HPV type	
16,18	17(54.8%)
Others	8(25.8%)
Negative	6(19.4%)
TMB	
Median (range)	6.2(2.1-137)
CIN	
Median (range)	0.24(0.009-0.57)
Best response	
CR	5(16.1%)
PR	12(38.7%)
SD	7(22.6%)
NE	7(22.6%)

HPV others include HPV 31,35,58,59,68,81.

Abbreviations: CIN, chromosomal instability; CR, complete response; FIGO, the Federation of Gynecology and Obstetrics; HPV, human papillomavirus; NE, not evaluated; PR, partial response; SD, stable disease; TMB, tumor mutation burden.

The overall maturity of survival data reached 38.7% (12/31), with 12 patients experienced disease progression during the follow-up period. Treatment responses included 5 complete responses (CR, 16.1%), 12 partial responses (PR, 38.7%), and 7 cases of stable disease (SD, 22.6%). The median DFS for the cohort was 19.6 months (95% CI: 14.7-NR).

### Genomic characteristics of the study cohort

**Figure 1.**
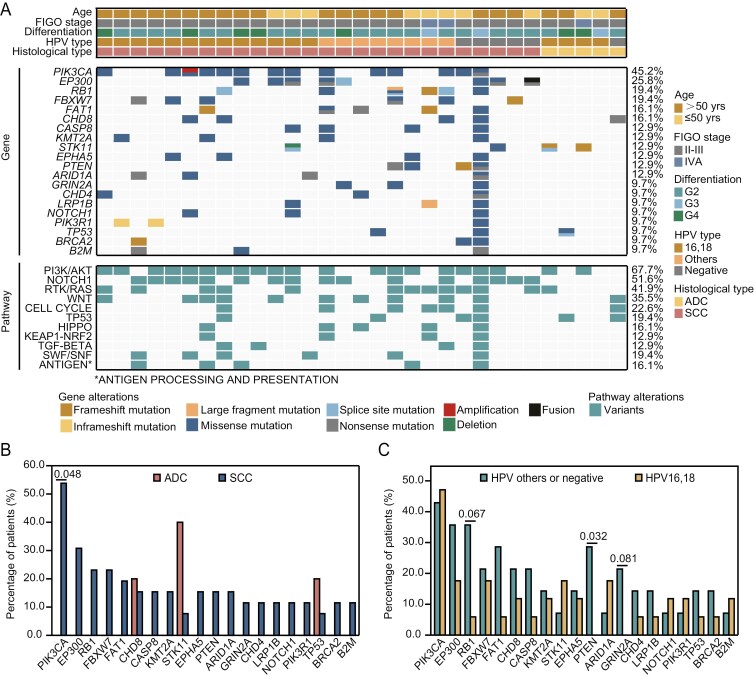
Genomic landscape of patients with unresectable cervical cancer. (A) The distribution of the most frequently mutated genes and oncogenic pathways in each patient. Clinical characteristics of each patient are displayed at the top. (B) Comparison of mutation frequencies of the top mutated cancer-associated genes between patients infected with SCC (*n* = 26) and ADC (*n* = 5). (C) Comparison of mutation frequencies of the top mutated cancer-associated genes between patients infected with high-risk HPV 16/18 (*n* = 17) and those with other HPV types or negative (*n* = 14). Note: other HPV types include HPV 31,35,58,59,68,81. Abbreviations: ADC, adenocarcinoma; FIGO, the Federation of Gynecology and Obstetrics; HPV, human papillomavirus; SCC, squamous cell carcinoma; yrs, years.

Genomic profiling of all patient samples was performed using targeted NGS to characterize the mutational landscape of cervical cancer within our cohort. The overall findings align with results from previous genomic studies on cervical cancer.^30^ The most frequently mutated gene was *PIK3CA*, observed in 45.2% of the patients, followed by *EP300* (25.8%), *RB1* (19.4%), *FBXW7* (19.4%), and *FAT1* (16.1%) (Figure 1A). Given that *PIK3CA* is a key component of the PI3K/AKT pathway, it is not surprising that examinations of alterations in 10 classic cancer-associated pathways^31^ revealed that this pathway as the most frequently altered, affecting 67.7% of the patients (Figure 1A).

Further subgroup comparisons revealed that mutations in *PIK3CA* were exclusively detected in patients with SCC, showing significant enrichment compared to those with ADC (53.8%, *P* = 0.048, Figure 1B). Although mutations in several other genes, including *EP300*, *RB1*, and *FBXW1*, were also found exclusively in SCC cases, no statistically significant differences were observed between the 2 histological subtypes (Figure 1B), likely due to the small sample size of the ADC subgroup. In addition, HPV infection status was found to correlate with specific genetic alterations (Figure 1C). Notably, mutations in *PTEN* (28.6% vs 0%, P = 0.032) and *GRIN2A* (21.4% vs 0%, *P* = 0.081) were absent in HPV16/18-positive patients but enriched among patients with other HPV types or HPV-negative status (Figure 1C). There was also a trend toward fewer HPV16/18-positive patients harboring *RB1* mutations compared to those with other HPV types or negative HPV status (5.9% vs. 35.7%, *P* = .067, Figure 1C).

### Predictive biomarkers of dCRT efficacy

**Figure 2.**
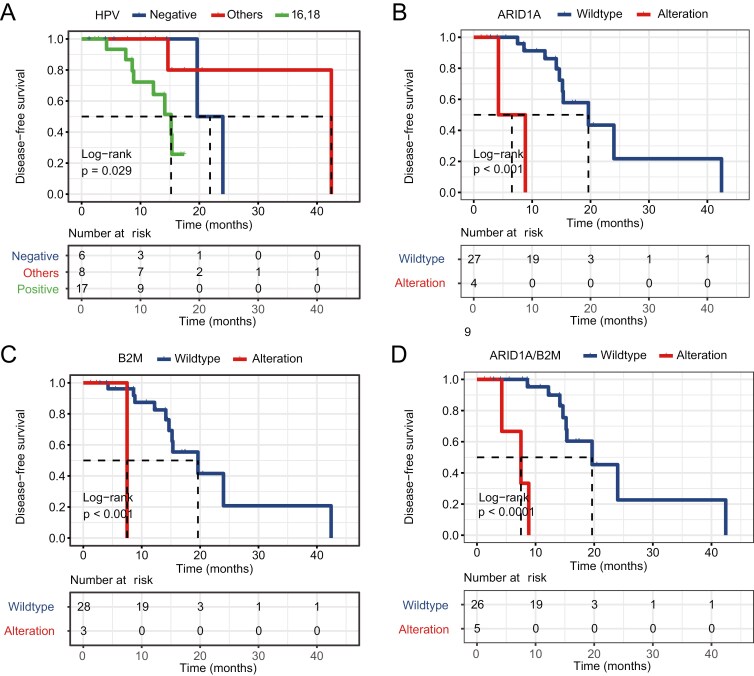
Associations of molecular features with survival following dCRT. (A) Kaplan-Meier estimates of DFS comparing patients with high-risk HPV 16/18, other HPV types, and HPV-negative status. (B) Kaplan-Meier estimates of DFS comparing patients with and without *ARD1A* alterations. (C) Kaplan-Meier estimates of DFS comparing patients with and without *B2M* alterations. (D) Kaplan-Meier estimates of DFS comparing patients with and without both *ARID1A/B2M* alterations. Other HPV types include HPV 31,35,58,59,68,81. Abbreviations: dCRT, definitive chemoradiotherapy; DFS, disease − free survival; HPV, human papillomavirus.

To investigate potential predictors of dCRT efficacy, we analyzed DFS in relation to clinical and genomic features. Consistent with HPV16/18 being the high-risk type, HPV16/18-positive patients exhibited shorter DFS compared to those with other HPV types or negative HPV status (*P* = .029; Supplementary Table S1 and Figure 2A). No significant associations between DFS and other clinical factors, such as age, FIGO stage, histological subtype, and degree of differentiation, were observed (Supplementary Table S1 and Figure S1).

We further evaluated the relationship between specific gene mutations and DFS outcomes. *PIK3CA* was the most frequently mutated gene in our cohort; however, its mutation status was not significantly associated with DFS (HR = 1.75, 95% CI = 0.50-6.16, *P* = .375; Supplementary Table S1). In contrast, patients with *ARID1A* mutations had significantly shorter DFS compared to those with the wildtype gene (HR = 18.86, 95% CI = 2.62-135.88, *P* < 0.001; Supplementary Table S1 and Figure 2B). Notably, among the 4 patients with *ARID1A* mutations in our cohort, 3 had nonsense mutations (Supplementary Figure S2A). Mutations in *B2M* were also associated with a poor DFS outcome (HR = 24.98, 95% CI = 1.56-399.59, *P* < .001; Supplementary Table S1 and Figure 2C). Among the 3 patients with *B2M* mutations, one presented with a nonsense mutation, another carried a frameshift mutation, and the third had a missense mutation of unclear functional significance (Supplementary Figure S2B).

At the pathway level, alterations in the classic cancer-associated pathways,^31^ including the PI3K/AKT pathway, showed no significant associations with DFS (Supplementary Table S1). However, given the association of *ARID1A* and *B2M* with poor DFS outcomes, we further investigated their corresponding pathways, the SWI/SNF pathway and the antigen processing and presentation pathway, respectively. We found that the alterations in the antigen processing and presentation pathway were significantly associated with shorter DFS (HR = 6.36, 95% CI = 1.13-35.93, *P* = 0.017; Supplementary Figure S3B). In contrast, the impact of SWI/SNF pathway alterations on DFS did not reach statistical significance (HR = 1.58, 95% CI = 0.40-6.27, *P* = 0.513; Supplementary Figure S3A).

We also evaluated TMB and CIN for their predictive potential in dCRT efficacy, but neither metric showed significant correlations with DFS across various cutoff values (Supplementary Figure S4A and S4B).

Multivariate analysis confirmed that *ARID1A* (HR = 26.24, 95% CI = 2.30–299.88, *P* = .009) and *B2M* (HR = 53.94, 95% CI = 2.05-1421.98, *P* = .017) mutations served as independent, negative predictors of dCRT efficacy (Supplementary Table S1). In contrast, HPV16/18 positivity was not independently associated with DFS outcomes in multivariate analysis (HR = 2.28, 95% CI = 0.67-7.91, *P* = 0.188; Supplementary Table S1). Notably, the concurrent presence of *ARID1A* and *B2M* mutations yielded the strongest prognostic impact, demonstrating markedly poorer DFS for patients harboring both mutations compared to wild-type (HR = 43.80, 95% CI = 4.41-434.50, *P* < 0.001; Supplementary Table S1 and Figure 2D).

### Suppressed immune microenvironment in patients with ARID1A/B2M gene mutations

**Figure 3.**
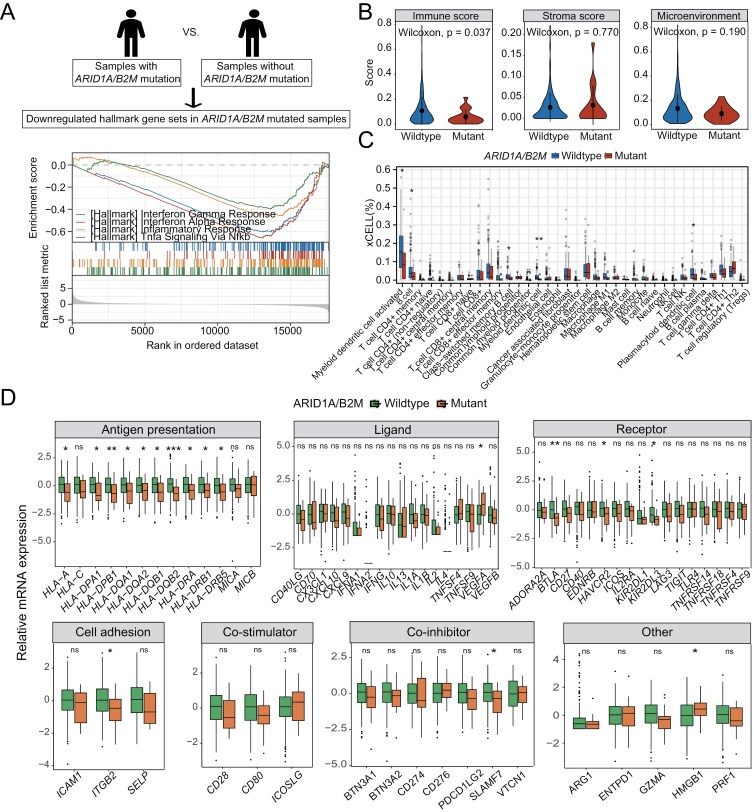
Expression analysis comparing *ARIDIA/B2M*-mutant versus wild-type samples in TCGA-CCA cohort. (A) GSEA of 50 hallmark gene sets comparing *ARIDIA/B2M*-mutant versus wild-type sample sets. Gene sets were obtained from MSigDB. (B) Violin plot of immune, stroma, and microenvironment score between *ARID1A/B2M*-mutated and *ARID1A/B2M*-wildtype samples based on the xCell algorithm. (C) Boxplot of immune infiltration levels between *ARID1A/B2M*-mutated and wild-type samples based on the xCell algorithm (* *P* < .05, ** *P* < .01, and *** *P* < .001). (D) Relative expression of immune-related genes in *ARID1A/B2M*-mutated and wild-type samples (* *P* < .05, **P < .01, and *** *P* < .001). Abbreviations: GSEA, gene set enrichment analysis; ns, no significance; TCGA-CCA, The Cancer Genome Atlas Cervical Cancer.

Both *ARID1A* and *B2M* deficiencies have been implicated in the dysregulation of the immune system.^32-34^ This prompted us to explore their effects on the immune microenvironment to uncover mechanisms that may influence dCRT outcomes. We conducted GSEA on an external cohort of 306 patients from the TCGA-CCA database harboring *ARID1A/B2M* mutations. Consistent with their role in immune response, the presence of *ARID1A* and *B2M* mutations were associated with the downregulation of several immune-related pathways (FDR < 0.25, adjusted *P* < .05; Supplementary Table S2). These downregulated pathways included the interferon response, inflammatory response, and NFkB signaling pathways (Figure 3A), which collectively suggest an immune-compromised state. Supporting this observation, the immune score calculated using xCell^25^ was significantly lower in *ARID1A/B2M*-mutant samples compared to wild-type samples (*P* = .037; Figure 3B), indicating reduced immune cell infiltration. No clear differences were observed in the stroma score (*P* = .770; Figure 3B) or the overall microenvironment score (*P* = .190; Figure 3B) comparing *ARID1A/B2M*-mutant or wildtype samples.

We focused on changes in specific immune cell populations. Notably, mutations in *ARID1A/B2M* were associated with reduced infiltration of myeloid dendritic cells, which are antigen-presenting cells that facilitate immune response.^35,36^ In addition, we observed decreased levels of B cells and plasmacytoid dendritic cells, the latter being responsible for the production of type I interferons^37^ (Figure 3C). These findings suggest that a compromised immune microenvironment that could hinder the effectiveness of dCRT in *ARID1A/B2M*-mutant patients.

We further examined immune-related gene expressions in *ARID1A/B2M*-mutant samples. In line with an impaired capacity for antigen presentation, there was notable downregulation of several key genes within the antigen presentation pathway, particularly among the human leukocyte antigen (HLA) genes, including *HLA-A*, *HLA-DPA1*, *HLA-DPB1*, and *HLA-DQA1* (Figure 3D). This pattern suggests a potential compromise in antigen presentation, a critical function for initiating adaptive immune responses.^38^ Aside from this, few differences were observed in other biological categories, except for an increased expression of *VEGFA* (Figure 3D). As a key mediator of angiogenesis, the upregulation of *VEGFA* may contribute to a pro-tumor microenvironment by enhancing tumor vascularization.^39^

### Potential impact of alterations in the antigen processing and presentation pathway

**Figure 4.**
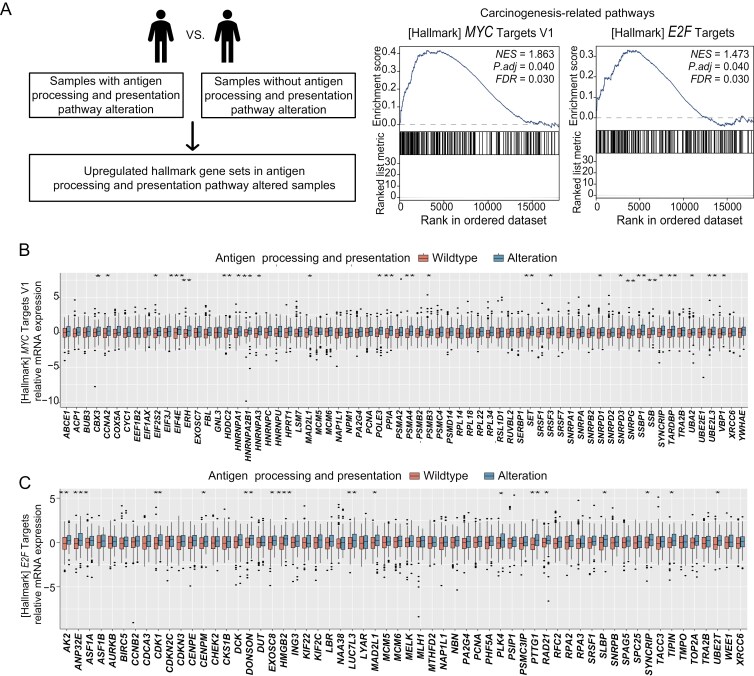
Expression analysis comparing antigen processing and presentation pathway-altered versus wild-type samples in TCGA-CCA cohort. (A) GSEA of 50 hallmark gene sets comparing pathway-altered versus wild-type sample sets. Gene sets were obtained from MSigDB. (B) Relative expression of MYC Targets V1 pathway genes in pathway-altered versus wild-type samples (*P < .05, **P < .01, and *** *P* < .001). (C) Relative expression of E2F Targets pathway genes in pathway-altered versus wild-type samples (*P < .05, **P < .01, and *** *P* < .001). Abbreviations: FDR, false discovery rate; GSEA, gene set enrichment analysis; NES, normalized enrichment score; ns, no significance; TCGA-CCA, The Cancer Genome Atlas Cervical Cancer.

Our findings highlight the critical role of dysregulated antigen processing and presentation pathway in contributing to the unfavorable DFS observed in cervical cancer patients following dCRT. This association was evident at both the DNA and RNA levels, leading us to further investigate the effects of such alterations on patient outcomes. To assess this, we performed GSEA on RNA-seq data from the TCGA-CCA database, focusing on samples with and without alterations in the antigen processing and presentation pathway (Figure 4A). Our analysis revealed significant upregulation of 2 key carcinogenesis-related pathways, MYC Targets V1 and E2F Targets, in samples with alterations in the antigen processing and presentation pathway (MYC targets V1: FDR = 0.030, adj. *P* = .040; E2F Targets: FDR = 0.030, adj. *P* = .040; Figure 4A and Supplementary Table S3). We subsequently assessed the expression levels of genes^26,27^ within the MYC Targets V1 and E2F Targets pathways, comparing samples with and without these antigen processing and presentation pathway gene alterations. Specifically, we identified 26 genes in the MYC Targets V1 pathway, including *CBX3* and *CCNA2*, that were significantly upregulated in the altered samples (Figure 4B). Similarly, in the E2F Targets pathway, we identified 16 oncogenesis-related genes, such as *AK2* and *ANP32E*, that exhibited elevated expression in the altered samples (Figure 4C). These findings suggest that the poor response to dCRT in patients with alterations in the antigen processing and presentation pathway may be attributed to the activation of these oncogenic pathways, potentially contributing to enhanced cancer progression and reduced treatment efficacy.

## Discussion

In this study, we conducted a comprehensive analysis of 31 patients with LACC undergoing dCRT. Our findings elucidate the clinical and genomic characteristics of these patients, shedding light on significant predictive biomarkers, including *ARID1A*, *B2M* and the antigen processing and presentation pathway, for treatment outcomes following dCRT. Our genomic analysis revealed a mutational landscape consistent with prior reports,^17,30,40^ with frequent mutations in *PIK3CA*, *EP300*, *RB1*, *FBXW7*, and *FAT1*. Notably, *PIK3CA* mutations emerged as the most prevalent among the cohort, highlighting its role in cervical cancer pathogenesis.^41,42^ The significant enrichment of *PIK3CA* mutations in SCC cases suggests a possible histological specificity in mutation patterns, warranting a tailored therapeutic approach. In addition, we observed PI3K/AKT pathway alterations in 67.7% of the cohort, a finding that is consistent with other studies,^43^ underscores the clinical relevance of this pathway in disease progression. The correlation between HPV status and genetic alterations further indicates that HPV16/18-positive patients, associated with a higher risk and poorer prognosis, experience distinct genomic profiles compared to those harboring other high-risk HPV types or no HPV at all.

Importantly, our analysis of predictive biomarkers of dCRT efficacy highlighted *ARID1A* and *B2M* mutations as significant negative predictors of DFS. *ARID1A* is a component of the SWI/SNF chromatin remodeling complex, plays an important role in gene regulation and chromatin structure.^32,44,45^  *B2M* is important for antigen processing and presentation via MHC class I molecules. Its dysfunction can impair immune surveillance, potentially allowing cancer cells to evade immune detection.^33^ Our results show that *ARID1A* and *B2M* mutations are each associated with significant short DFS, with the combined presence of these mutations linked to an even more pronounced decrease in survival, indicating a possible synergistic effect. The dramatic reduction in DFS observed in patients with these mutations reinforces the need for routine genomic screening to guide clinical decisions.

In further exploring the immune microenvironment, our findings reveal substantial immune suppression in patients with *ARID1A/B2M* mutations. We observed downregulation of immune-related pathways, including interferon and inflammatory signaling, accompanied by a significant reduction in immune scores (P = 0.037), suggesting a compromised immune response. Moreover, *ARID1A/B2M* mutant samples exhibited lower infiltration of key antitumor immune cells, such as myeloid dendritic cells, B cells, and class-switched memory B cells, indicating diminished immune surveillance within the tumor microenvironment.^38^ The downregulation of critical immune pathways, including those involved in antigen presentation via downregulation of *HLA* genes^38^ may further explain the compromised efficacy of dCRT in these patients. These immune deficiencies may underline the poor DFS observed in dCRT-treated *ARID1A/B2M*-mutant patients.

Notably, alterations in the antigen processing and presentation pathway were linked to prominent oncogenic signaling, as evidenced by the upregulation of MYC Targets V1 and E2F Targets pathways. These upregulations suggest a potential mechanistic link between the dysregulation of antigen processing and presentation pathway and cancer progression. Specifically, aberrations in this pathway may facilitate cancer development by activating oncogenic signaling cascades. *MYC* is a well-known oncogene involved in regulating processes critical to tumorigenesis, such as angiogenesis, cell adhesion, metabolism, and immune evasion, thereby promoting malignancy and tumor aggression.^46,47^ Our finding suggests that alterations in the antigen processing and presentation pathway may accelerate cancer progression by activating oncogenic pathways.^48^ This connection underscores the potential for aberrations in immune-related pathways to influence not just tumor biology but also therapeutic responses. The identification of key oncogenes within the MYC and E2F pathways, such as *CBX3* and *AK2*, highlights specific targets for further investigation.

One limitation of this study is the relatively small sample size and its retrospective nature, which may affect the generalizability of our findings. Furthermore, the lack of functional validation for the identified biomarkers highlights the need for future research to confirm these associations.

Another limitation of our study is the exclusion of patients over 70 years old and those with severe or uncontrolled comorbidities. These criteria were implemented to minimize potential confounding factors that could influence DFS, particularly those affecting treatment tolerance. However, we acknowledge that this exclusion limits the generalizability of our findings. Elderly patients and those with comorbidities represent a significant portion of the cervical cancer population and may benefit substantially from biomarker-driven therapies. Future studies should aim to include these groups to evaluate whether the identified biomarkers, such as *ARID1A* and *B2M* mutations, can inform treatment decisions and improve outcomes across a more diverse patient population.

Future prospective studies with larger cohorts are crucial to substantiate our results, particularly regarding the mechanistic roles of *ARID1A* and *B2M* mutations. Moreover, exploring combination therapies that include immune checkpoint inhibitors or other immunomodulatory agents could provide promising strategies to enhance treatment response in patients with *ARID1A/B2M* mutations. Given the compromised immune microenvironment observed in these patients, immunotherapy-based approaches may potentially improve clinical outcomes and address the limitations of dCRT in this subset of cervical cancer patients.

In conclusion, our study affirms the complex interplay between genomic alterations, immune evasion, and treatment outcomes in cervical cancer. Our findings provide valuable insights into the genetic and immunological factors influencing dCRT response in cervical cancer. The identification of *ARID1A* and *B2M* mutations, particularly in the context of antigen procession and presentation pathway, as key predictors of poor DFS offers a compelling rationale for personalized treatment strategies aimed at improving outcomes in this patient populations. Future work should focus on validating these findings in larger cohorts and exploring targeted therapeutic interventions that address both the genomic and immunological complexities of cervical cancer.

## Supplementary Material

oyaf133_suppl_Supplementary_Figures_S1-S4

oyaf133_suppl_Supplementary_Tables_S1

oyaf133_suppl_Supplementary_Tables_S2

oyaf133_suppl_Supplementary_Tables_S3

## Data Availability

The datasets generated and analyzed during this current study are available from the corresponding author upon reasonable request.
