# Supplementary material for: Predictive role of ARID1A and B2M mutations and the antigen presentation pathway in the efficacy of definitive chemoradiotherapy for cervical cancer
Source: Oncologist. 2025 Jun 19;30(6):oyaf133. doi: 10.1093/oncolo/oyaf133 (PMC12204396; doi:10.1093/oncolo/oyaf133)
Supplement: oyaf133_suppl_Supplementary_Figures_S1-S4 [file oyaf133_suppl_supplementary_figures_s1-s4.zip › Figure S1.pdf]

FigureS1

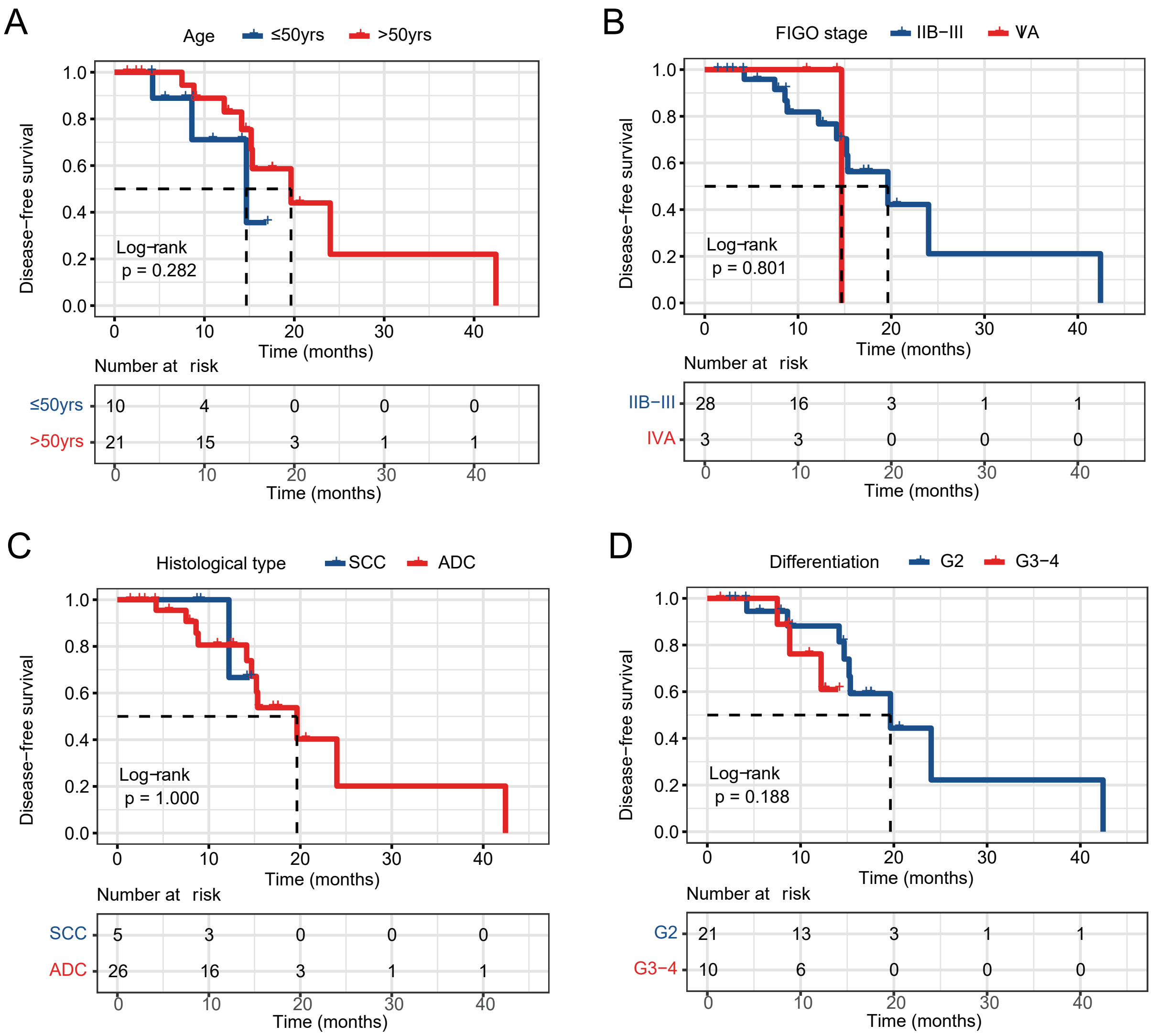
A
Age ≤50yrs >50yrs
Disease−free survival
Log-rank
p = 0.282
Time (months)
Number at risk
≤50yrs 10 4 0 0 0
>50yrs 21 15 3 1 1
B
FIGO stage IIB−III IVA
Log-rank
p = 0.801
Number at risk
IIB−III 28 16 3 1 1
IVA 3 3 0 0 0
C
Histological type SCC ADC
Log-rank
p = 1.000
Number at risk
SCC 5 3 0 0 0
ADC 26 16 3 1 1
D
Differentiation G2 G3−4
Log-rank
p = 0.188
Number at risk
G2 21 13 3 1 1
G3−4 10 6 0 0 0
