# Supplementary material for: Predictive role of ARID1A and B2M mutations and the antigen presentation pathway in the efficacy of definitive chemoradiotherapy for cervical cancer
Source: Oncologist. 2025 Jun 19;30(6):oyaf133. doi: 10.1093/oncolo/oyaf133 (PMC12204396; doi:10.1093/oncolo/oyaf133)
Supplement: oyaf133_suppl_Supplementary_Figures_S1-S4 [file oyaf133_suppl_supplementary_figures_s1-s4.zip › Supplementary Figure legends.docx]

Figure S1. Kaplan-Meier survival curves for the disease-free survival of patients stratified by clinical characteristics at diagnosis: (A) age groups, (B) the FIGO stage, (C) histological type, and (D) differentiation grade. ADC, adenocarcinoma; FIGO, the Federation of Gynecology and Obstetrics; SCC, squamous cell carcinoma; yrs, years.

Figure S2. Lollipop plots illustrating somatic mutations in (A) *ARID1A* and (B) *B2M*, mapped to their respective protein domains.

Figure S3. Kaplan-Meier survival curves for the disease-free survival of patients with and without alterations in the (A) SWI/SNF pathway and (B) Antigen Processing and Presentation pathway.

Figure S4. Associations of TMB and CIN survival following dCRT. (A) Forest plot for TMB. (B) Forest plot for CIN. CI, confidence interval; CIN, chromosomal instability; dCRT, definitive chemoradiotherapy; HR, hazard ratio; TMB, tumor mutation burden.
