# Supplementary figures and images for: Predictive role of ARID1A and B2M mutations and the antigen presentation pathway in the efficacy of definitive chemoradiotherapy for cervical cancer
Source: Oncologist. 2025 Jun 19;30(6):oyaf133. doi: 10.1093/oncolo/oyaf133 (PMC12204396; doi:10.1093/oncolo/oyaf133)

A

ARID1A

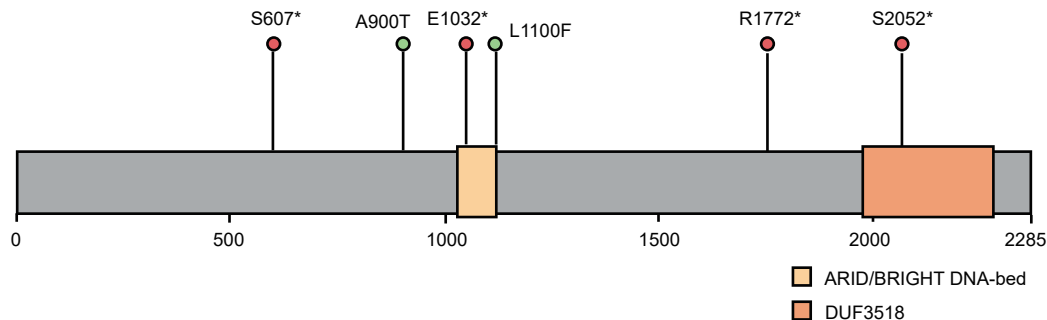

B

B2M

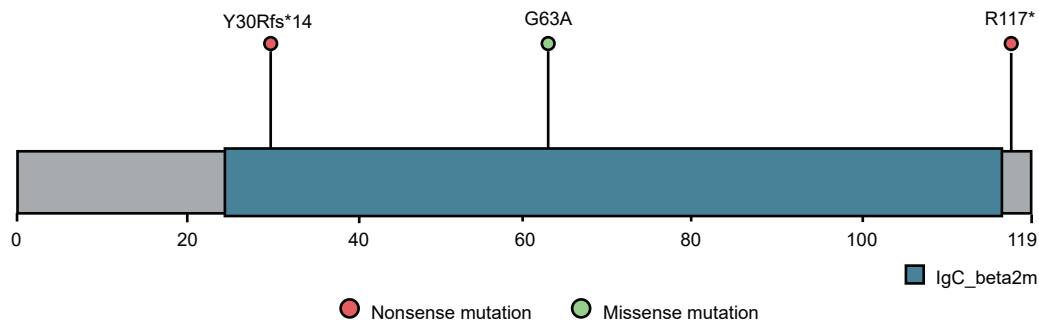

Supplement: oyaf133_suppl_Supplementary_Figures_S1-S4 [file oyaf133_suppl_supplementary_figures_s1-s4.zip › Figure S2.pdf]

Figure S3

A

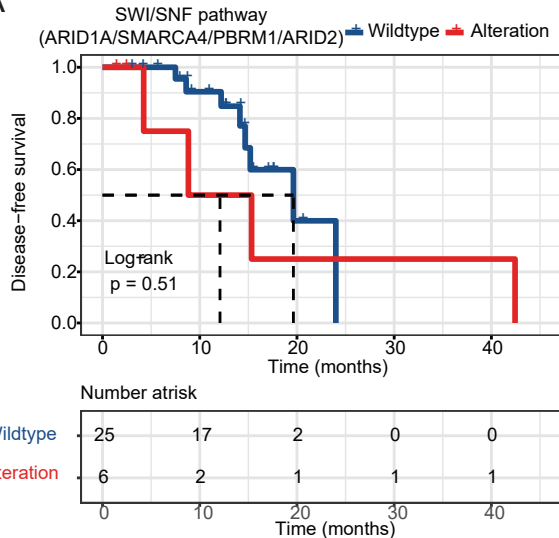

B

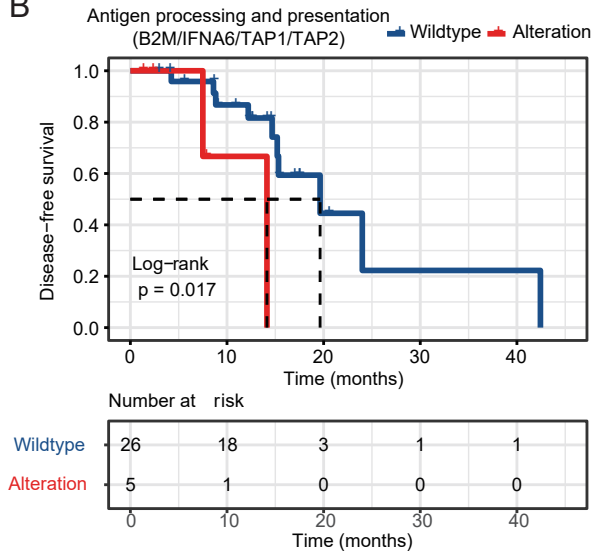

Supplement: oyaf133_suppl_Supplementary_Figures_S1-S4 [file oyaf133_suppl_supplementary_figures_s1-s4.zip › Figure S3.pdf]

A

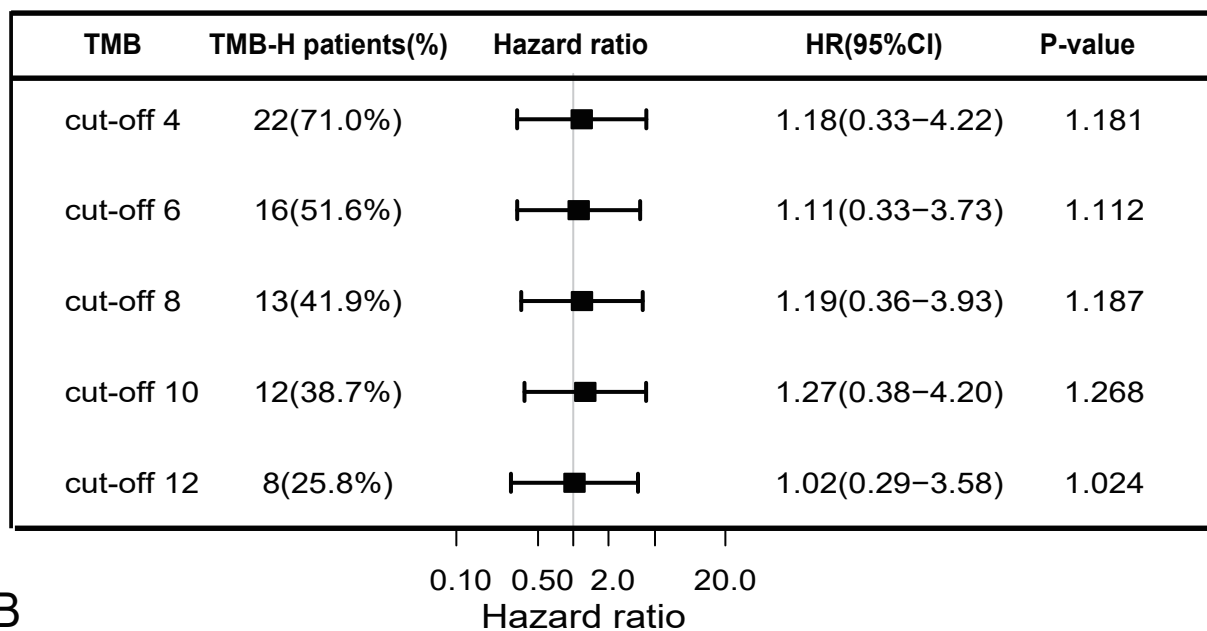

B

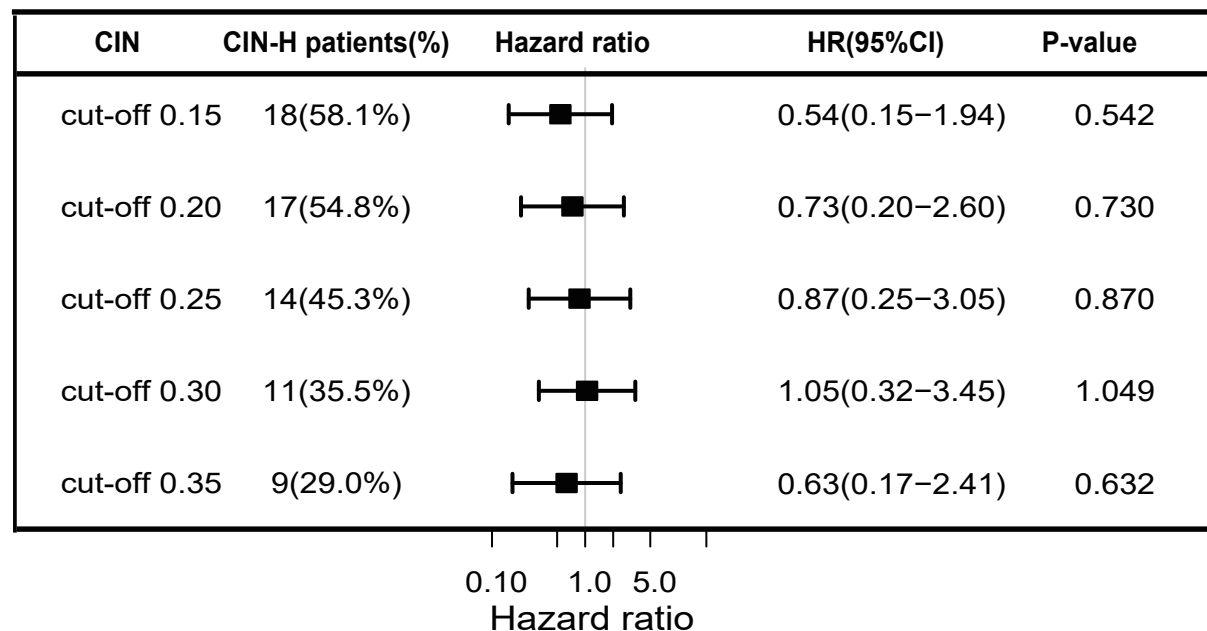

Supplement: oyaf133_suppl_Supplementary_Figures_S1-S4 [file oyaf133_suppl_supplementary_figures_s1-s4.zip › Figure S4.pdf]
