# Supplementary material for: Predictive role of ARID1A and B2M mutations and the antigen presentation pathway in the efficacy of definitive chemoradiotherapy for cervical cancer
Source: Oncologist. 2025 Jun 19;30(6):oyaf133. doi: 10.1093/oncolo/oyaf133 (PMC12204396; doi:10.1093/oncolo/oyaf133)
Supplement: oyaf133_suppl_Supplementary_Tables_S1 [file oyaf133_suppl_supplementary_tables_s1.docx]

**Table S1.** **Univariate and multivariate cox regression analyses of risk factors**.

| Factors | Univariate analysis | | Multivariate analysis | |  |
| --- | --- | --- | --- | --- | --- |
|  | HR (95% CI) | P-value | HR (95% CI) | P-value |  |
| Clinical characteristics | | | | |  |
| Age (＞50yrs vs. ≤50yrs) | 0.47(0.11-1.93) | 0.282 |  |  |  |
| FIGO stage (IVA vs. IIB-III) | 1.31(0.16-10.98) | 0.801 |  |  |  |
| Differentiation (G3-4 vs. G1-2) | 3.14(0.52-18.83) | 0.188 |  |  |  |
| HPV (16,18 vs. others vs. negative) | - | **0.029** | 2.28(0.67-7.79) | 0.188 |  |
| Histological type (SCC vs. ADC) | 1.00(0.12-8.69) | 1.000 |  |  |  |
| Gene (Alteration vs. wildtype) | | | | |  |
| *PIK3CA* | 1.75(0.50-6.16) | 0.375 |  |  |  |
| *EP300* | 1.27(0.33-4.94) | 0.729 |  |  |  |
| *RB1* | 0.55(0.12-2.60) | 0.442 |  |  |  |
| *FBXW7* | 1.05(0.26-4.20) | 0.943 |  |  |  |
| *FAT1* | 0.46(0.06-3.66) | 0.454 |  |  |  |
| *CHD8* | 2.59(0.64-10.50) | 0.166 |  |  |  |
| *CASP8* | 4.24E-09(0-Inf) | 0.217 |  |  |  |
| *KMT2A* | 0.62(0.08-4.97) | 0.646 |  |  |  |
| *STK11* | 0.49(0.06-3.85) | 0.485 |  |  |  |
| *EPHA5* | 1.92(0.39-9.37) | 0.412 |  |  |  |
| *PTEN* | 3.25E-09(0-Inf) | 0.165 |  |  |  |
| *ARID1A* | 18.86(2.62-135.88) | **<0.001** | 26.24(2.30-299.877) | **0.009** |  |
| *GRIN2A* | 3.34E-09(0-Inf) | 0.147 |  |  |  |
| *CHD4* | 4.46E-09(0-Inf) | 0.265 |  |  |  |
| *LRP1B* | 1.31E-08(0-Inf) | 0.356 |  |  |  |
| *NOTCH1* | 1.33(0.17-10.66) | 0.789 |  |  |  |
| *PIK3R1* | 1.34E-08(0-Inf) | 0.418 |  |  |  |
| *TP53* | 1.23(0.15-9.86) | 0.845 |  |  |  |
| *BRCA2* | NA(NA-NA) | 1.000 |  |  |  |
| *B2M* | 24.98(1.56-399.59) | **<0.001** | 53.94(2.05-1421.98) | **0.017** |  |
| Pathway (Alteration vs. wildtype) | | | | |  |
| PI3K/AKT | 1.14(0.29-4.46) | 0.847 |  |  |  |
| NOTCH_path | 1.87(0.48-7.34) | 0.364 |  |  |  |
| RTK/RAS | 0.67(0.18-2.54) | 0.552 |  |  |  |
| WNT | 1.02(0.31-3.37) | 0.969 |  |  |  |
| CELL CYCLE | 0.48(0.10-2.27) | 0.345 |  |  |  |
| TP53 | 1.20(0.25-5.78) | 0.824 |  |  |  |
| HIPPO | 0.46(0.06-3.66) | 0.454 |  |  |  |
| KEAP1-NRF2 | 0.92(0.11-7.37) | 0.937 |  |  |  |
| TGF-BETA | 1.82(0.38-8.84) | 0.449 |  |  |  |

Note: HPV others include HPV 31,35,58,59,68,81. Signiﬁcant P-values < 0.05 are bolded. Abbreviations: ADC, adenocarcinoma; CI, confidence interval; FIGO, the Federation of Gynecology and Obstetrics; HPV, high-risk human papillomavirus; HR, hazard ratio; NA, not applicable; SCC, squamous cell carcinoma.
