# Supplementary material for: Predictive role of ARID1A and B2M mutations and the antigen presentation pathway in the efficacy of definitive chemoradiotherapy for cervical cancer
Source: Oncologist. 2025 Jun 19;30(6):oyaf133. doi: 10.1093/oncolo/oyaf133 (PMC12204396; doi:10.1093/oncolo/oyaf133)
Supplement: oyaf133_suppl_Supplementary_Tables_S2 [file oyaf133_suppl_supplementary_tables_s2.docx]

**Table S2. Gene set enrichment analysis in *ARID1A/B2M*-mutated versus *ARID1A/B2M*-wildtype patients.**

| Gene set | NES | p.adjust | qvalue |
| --- | --- | --- | --- |
| HALLMARK_INTERFERON_GAMMA_RESPONSE | -2.332 | <0.001 | <0.001 |
| HALLMARK_ALLOGRAFT_REJECTION | -2.331 | <0.001 | <0.001 |
| HALLMARK_INTERFERON_ALPHA_RESPONSE | -2.297 | <0.001 | <0.001 |
| HALLMARK_INFLAMMATORY_RESPONSE | -1.763 | <0.001 | <0.001 |
| HALLMARK_COMPLEMENT | -1.584 | 0.004 | 0.003 |
| HALLMARK_TNFA_SIGNALING_VIA_NFKB | -1.530 | 0.007 | 0.006 |
| HALLMARK_P53_PATHWAY | -1.496 | 0.007 | 0.006 |

Abbreviation: NES, normalized enrichment score; p.adjust, adjusted *P-value*.
