# Supplementary material for: Predictive role of ARID1A and B2M mutations and the antigen presentation pathway in the efficacy of definitive chemoradiotherapy for cervical cancer
Source: Oncologist. 2025 Jun 19;30(6):oyaf133. doi: 10.1093/oncolo/oyaf133 (PMC12204396; doi:10.1093/oncolo/oyaf133)
Supplement: oyaf133_suppl_Supplementary_Tables_S3 [file oyaf133_suppl_supplementary_tables_s3.docx]

**Table S3. Gene set enrichment analysis in antigen presentation pathway-altered versus pathway-wildtype patients.**

| Gene set | NES | p.adjust | qvalue |
| --- | --- | --- | --- |
| HALLMARK_WNT_BETA_CATENIN_SIGNALING | -2.110 | 0.001 | 0.001 |
| HALLMARK_OXIDATIVE_PHOSPHORYLATION | 2.313 | 0.040 | 0.030 |
| HALLMARK_MYC_TARGETS_V1 | 1.863 | 0.040 | 0.030 |
| HALLMARK_E2F_TARGETS | 1.473 | 0.040 | 0.030 |
| HALLMARK_FATTY_ACID_METABOLISM | 1.411 | 0.044 | 0.034 |

Abbreviation: NES, normalized enrichment score; p.adjust, adjusted *P-value*.
